# Protein Kinase A and Anxiety-Related Behaviors: A Mini-Review

**DOI:** 10.3389/fendo.2016.00083

**Published:** 2016-06-29

**Authors:** Margaret F. Keil, George Briassoulis, Constantine A. Stratakis, T. John Wu

**Affiliations:** ^1^Section on Endocrinology and Genetics, Eunice Kennedy Shriver National Institute of Child Health and Human Development (NICHD), National Institutes of Health (NIH), Bethesda, MD, USA; ^2^Department of Pediatric Intensive Care, University of Crete, Heraklion, Greece; ^3^Department of Obstetrics and Gynecology, Center for Neuroscience and Regenerative Medicine, Uniformed Services University of the Health Sciences, Bethesda, MD, USA

**Keywords:** protein kinase A, anxiety, knockout mice, regulatory subunit, catalytic subunit

## Abstract

This review focuses on the anxiety related to cyclic AMP/protein kinase A (PKA) signaling pathway that regulates stress responses. PKA regulates an array of diverse signals that interact with various neurotransmitter systems associated with alertness, mood, and acute and social anxiety-like states. Recent mouse studies support the involvement of the PKA pathway in common neuropsychiatric disorders characterized by heightened activation of the amygdala. The amygdala is critical for adaptive responses leading to fear learning and aberrant fear memory and its heightened activation is widely thought to underpin various anxiety disorders. Stress-induced plasticity within the amygdala is involved in the transition from normal vigilance responses to emotional reactivity, fear over-generalization, and deficits in fear inhibition resulting in pathological anxiety and conditions, such as panic and depression. Human studies of PKA signaling defects also report an increased incidence of psychiatric disorders, including anxiety, depression, bipolar disorder, learning disorders, and attention deficit hyperactivity disorder. We speculate that the PKA system is uniquely suited for selective, molecularly targeted intervention that may be proven effective in anxiolytic therapy.

## Overview of the PKA Pathway

Protein kinase A (PKA) is an inactive tetrameric holoenzyme consisting of two catalytic (C) subunits each bound to a regulatory (R) subunit dimer. The four R subunits RIα, RIβ, RIIα, and RIIβ, coded by different genes, characterize the subtypes PKA-I and -II (1). Different genes also code for the four catalytic (C) subunits Cα, Cβ, Cγ, and protein kinase X (PRKX), which are expressed (like the R subunits) in a cell- and tissue-specific manner (2). When the R and C subunits form the PKA tetramer (R2C2), there is no PKA catalytic activity. Additionally, R2C2 is bound and, thus, compartmentalized within the cell by A-kinase anchoring proteins (AKAPs) that direct PKA-signaling to specific cell regions and/or organelles (3).

**Figure 1:**
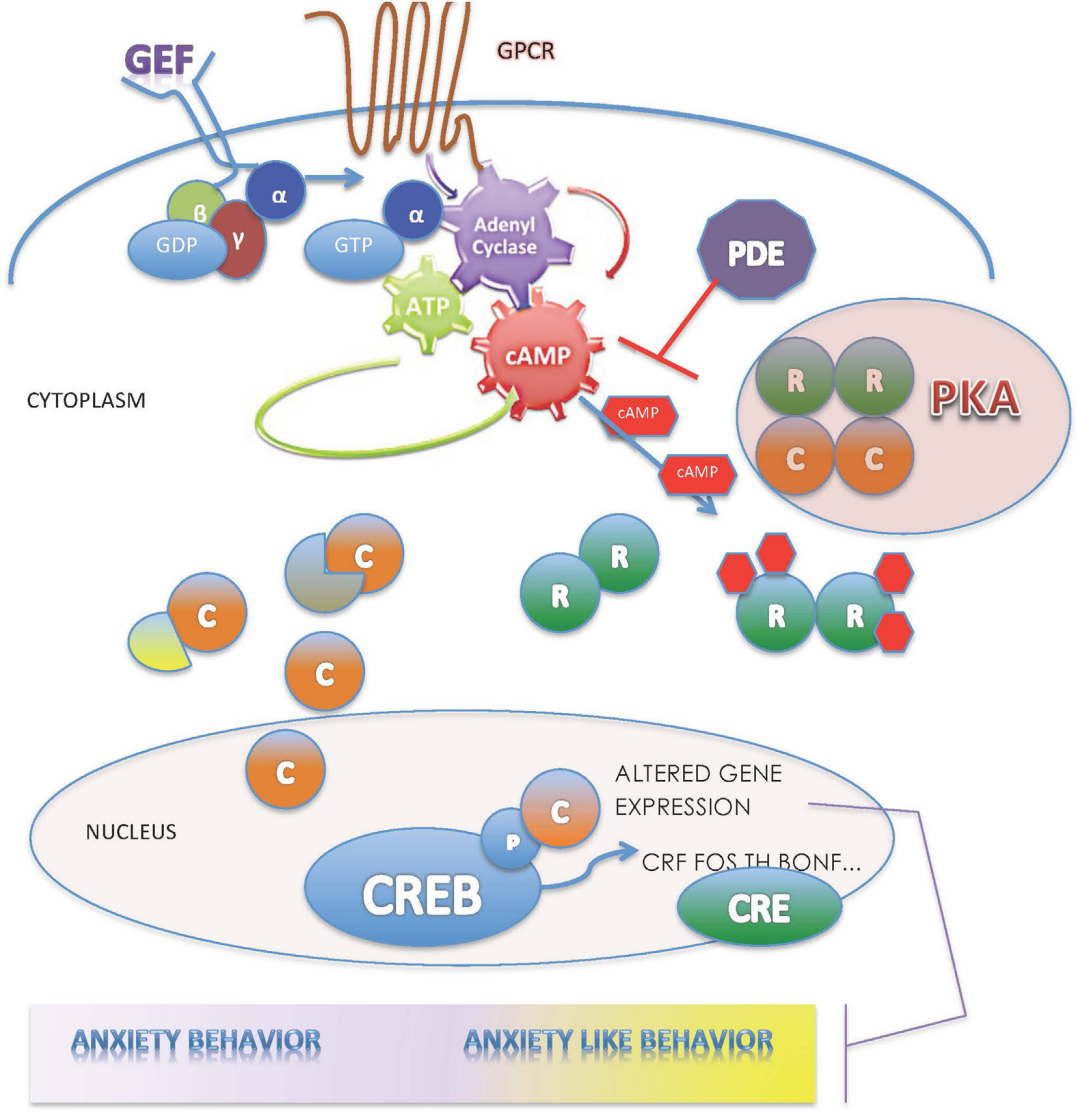
**The PKA enzyme is central in the regulation of the cAMP-signaling pathway; for description, please see the text**.

Protein kinase A is considered the primary target for cyclic AMP (cAMP) in the cell, is widely distributed, and serves as the principal effector mechanism for G-protein-coupled receptors (GPCRs) linked to adenylate cyclase (4). The seven-transmembrane domain GPCRs sense molecules outside the cell and activate inside signal transduction pathways to induce cellular responses. The majority of receptors for proteins, biogenic amines, protons, hormones, neurotransmitters, and neuromodulators elicit their responses through guanine nucleotide-binding proteins (G-proteins). The G-protein α subunits, encoded by 16 distinct genes, confer receptor–effector specificity to G-proteins. The γ subunits, encoded by 12 genes, have G protein-specific recognition sites and bind tightly to β subunits. The β subunits, encoded by five genes, also contain a common binding site for α subunit recognition. In their inactive state, the α and βγ subunits are bound to guanosine diphosphate (GDP) and can interact with effectors (Figure 1). This interaction releases GDP in an exchange with guanosine triphosphate (GTP) leading to the generation of α-GTP and a βγ subunit dimer (5, 6). Of the various Gα isoforms, the activation of G_s_α stimulates adenylyl cyclase resulting in the production of cAMP. By contrast, the Giα mediates the inhibition of adenylyl cyclase (7, 8).

The increased intracellular cAMP, serving as a second messenger, binds to the regulatory PKA subunits, leading to the disassociation of the tetrameric PKA holoenzyme into an R2-cAMP 4 dimer and two monomers of free catalytic subunits (9). Then, the PKA C subunits catalyze the transfer of phosphates from ATP to serine and threonine residues of targeted intracellular proteins modifying hormonal and neurotransmitter responses, desensitizing receptors related to cortisol biosynthesis, cell differentiation, and synaptic plasticity, and activating or repressing gene transcription (10–12). The principal target of C subunits that translocate to the nucleus is phosphorylation of cAMP-responsive nuclear factors (13) that regulate the expression of genes containing cAMP-responsive elements binding proteins (CREBs) (14) (Figure 1). Phosphorylated CREB binds to CRE nucleotide sequences in DNA as a dimer, recruiting CREB-binding protein (CBP) and p300 cofactors to form larger transcriptional complexes, and catalyzes histone acetylation regulating target genes. Activated CREBs coordinate various neuronal functions, development, and synaptic plasticity (15). Through a feedback mechanism, activated by RIα, cytosolic phosphodiesterases (PDEs) terminate the signals generated by cAMP by hydrolyzing cAMP into 5′AMP (16). As shown in knockout (KO) mouse studies, these four genes function in a tissue- and cell-type-specific manner to regulate accurately the activity of the C subunits (2).

## Corticotropin-Releasing Hormone with PKA

Corticotropin (ACTH)-releasing hormone (CRH)-induced intracellular increase in calcium and cAMP (and PKA activation) through binding to its seven-transmembrane receptor activates several transcription factors including CREB, c-fos, and JunB, which subsequently activate the pro-opiomelanocortin promoter (17). ιnnervation to CRH neurons is provided by fibers containing the pituitary adenylate cyclase-activating polypeptide (PACAP) (18). It is possible that stress induces secretion of PACAP in the paraventricular nucleus (PVN), thereby stimulating CRH gene expression via activation of the cAMP/PKA system (19). Especially, amygdala and lower brainstem contributions to the augmentation of the stress response have been identified as sites of pituitary PACAP innervation (19). Thus, CRH, released by stress signals, stimulates a pulsatile secretion of adrenocorticotropic hormone (ACTH) with peak levels seen before waking and declining at night.

Adrenocorticotropic hormone exerts its effects on the adrenal cortex by binding to a specific receptor (ACTHR) that is the melanocortin-2 receptor (MC2R, coded by the *MC2R* gene). MC2R is a GPCR linked to G_s_α and, thus, ACTH binding triggers activation of adenylate cyclase that catalyzes the conversion of adenosine triphosphate to cAMP (20). The elevation of cAMP is followed by increased PKA activity at the adrenal cortex that results in increased steroidogenesis and the production of glucocorticoids (GCs). Negative feedback on pituitary ACTH secretion is exerted by cortisol at both the hypothalamic and anterior pituitary levels. Importantly, hypothalamic cAMP-inducing CRH might also counterbalance excessive stimulatory stress effects on the hypothalamic–pituitary–adrenal (HPA) axis and maintain immuno-neuroendocrine homeostasis (21).

Other peptide hormones that are secreted and function in an endocrine manner also act as ligands and signal via a wide range of GPCRs. Such peptide hormones, mostly bind within the transmembrane domain, include the growth-regulating hormones somatostatins, parathyroid hormone, angiotensin, HCRTR2, oxytocin, calcitonin, C5a anaphylatoxin, cannabinoids, follicle-stimulating hormone, gonadotropin-releasing hormone, neurokinin, thyrotropin-releasing hormone, and the cholecystokinin peptide hormone system. GPCRs are responsive to hormones, calcium, and neurotransmitters allowing them to form the largest family of validated drug targets (22).

## Role of PKA in Anxiety and Fear Learning

This review focuses on the role of the cAMP-signaling pathway and its mediator, PKA, in the pathogenesis of disorders related to fear learning and anxiety. Anxiety disorders are associated with abnormalities in neural processing of threat-related stimuli (fear learning), which is regulated by cAMP/PKA pathway.

Preclinical studies provide evidence that the pathogenesis of mood disorders, such as anxiety and depression, involves alterations in the plasticity of neuronal pathways (23, 24). Also, clinical studies demonstrate that chronic stress and depression alter brain structures (i.e., cell number and density, cell body size, neuronal and glial density in frontal cortical and hypothalamus) and that result in functional changes (25–29). In addition, studies of suicide subjects report alterations in levels of serotonin and norepinephrine, their metabolites, and receptors, in the brain and peripheral tissues, as well as intracellular signaling pathways (30, 31). Post-mortem studies demonstrate disruptions in cAMP/PKA/CREB/Rap1/BDNF in the brains of suicide subjects that are modulated by stress and GCs (32). There is a paucity of data about PKA activity in brain areas other than the frontal cortex from post-mortem brains of depressed and suicidal subjects. The cAMP/PKA signaling pathway in the central nervous system is well-characterized and has a crucial role for various physiological responses that are important for cell survival, synaptic plasticity, and gene expression (33, 34). Alterations in synaptic and structural plasticity are associated with mood disorders, including generalized anxiety disorder, depression, and suicide.

### PKA and Fear Learning and Memory

There is substantial evidence from different species (fruit fly, mouse, chick, and rat) to support the role of cAMP/PKA signaling in the molecular pathways related to fear and fear memory. The seminal work of Schacher, Kandel, and Abel demonstrated the essential role of the cAMP/PKA pathway in the response to fear and consolidation of fear memory (15, 35, 36). Consistent with the evolutionarily conserved role of the cAMP/PKA pathway, anxiety and fear responses are essential to survival. The mechanisms of PKA in fear memory are well established and include a wide range of cellular processes, including activation of cAMP–CREB and various other transcription factors involved in the regulation of *de novo* protein synthesis that is required for long-term memory formation. Signaling activity in neural circuits pre- or post-stimuli may influence PKA activity and long-term potentiation (LTP), affecting fear learning and memory of the event (37–39). Also, there is a time-dependent activation profile in the kinase pathways involved in fear memory formation. PKA has two peaks of activity in the process of long-term memory formation, with the first occurring a few minutes after the event, and the second occurring 2 to 3 h after the event (requires both transcription and protein synthesis). The PKA pathway is also an important component of short-term memory within the first hour after the event. The phosphorylated form of CREB also increases at these same time periods as PKA and contributes to the synthesis of new proteins that are essential for long-term memory formation (40).

The cAMP–CREB element is ubiquitous in genes and functions as a promoter in many brain areas that respond to environmental stimuli. PKA signaling has been described as a “central hub” that interacts with varied other signaling pathways in neuroendocrine cells (41). PKA mediates and communicates cAMP effects to mitogen-activated protein kinases (MAPK) and protein kinase C (PKC) and B pathways. Signal transduction pathways, such as PKA and PKC, have important roles in the regulation of the HPA and autonomic nervous system (ANS) and, therefore, may have a role in the expression of genes that contain cAMP in their promoters, which include key proteins that regulate the neuroendocrine stress response (i.e., brain-derived neurotrophic factor and GC receptor) (42).

Types of traumatic stress, which have been associated with maladaptive responses or psychopathology, include mass trauma, war, terrorism, natural or technological disasters, violent personal assaults, child abuse (physical, sexual, emotional), life-threatening illness, and accidents. However, not every person who is exposed to traumatic stress will develop long-lasting psychological morbidity, such as depression, anxiety, or post traumatic stress disorder. The development and/or severity of these conditions depends on multiple factors, including genetic pre-disposition to vulnerability, exposure to adverse environmental factors, and the timing of the stress exposure (43, 44).

Typically, the stress response has been identified as a “fight or flight” reaction, but may also include an increased state of vigilance, which is often accompanied by increased anxiety. The response to an environmental stressor involves the individual’s interpretation of the threat, which is regulated by the brain. The brain and nervous system demonstrate adaptive plasticity through local neurotransmitters and systemic hormones, which interact to produce structural and functional changes (45).

The brain is also a target for the actions of stress hormones, in particular, GCs. With stress exposure, the PVN in the hypothalamus releases CRH and arginine vasopressin, which stimulate the anterior pituitary to release ACTH, which stimulates the adrenal cortex to release GCs. GCs exert a negative feedback to the hypothalamus and anterior pituitary to downregulate the stress response through their receptor (GR) which is found expressed highly in the hippocampus, amygdala, and prefrontal cortex. This facilitates the formation of memories associated with strong emotions particularly during stress.

### PKA and Anxiety

Anxiety is an adaptive response to a potential threat that serves a protective function. However, pathological anxiety is associated with abnormalities in fear learning or threat detection (46–49) and a bias to interpret ambiguous situations as threatening with corresponding behavioral responses of avoidance or exaggerated reactions to potential threats (50). Fear memories can form quickly and be difficult to eliminate (51, 52). Evidence from experimental and preclinical studies provides support that anxiety disorders are associated with abnormal neural processing of threat-related stimuli, which is mediated by the PKA pathway.

The amygdala, located in the temporal lobe of the brain, has a crucial role in the processing and expression of emotional stimuli (53, 54). Prior studies with humans and laboratory animals provide evidence that novelty and fear-related stimuli are both processed by the amygdala (55–58). Hyperactivity of the amygdala as demonstrated by functional neuroimaging studies in humans, has been identified as a neural correlate for clinical symptoms seen in post traumatic stress disorder (59, 60), which suggests that amygdala dysfunction may be a risk factor for development of affective stress-related disorders (61). The basolateral amygdala is identified as a hub through which sensory information is relayed either directly or indirectly via the basal nucleus to the central amygdala (CEA), which is the major efferent source that directs fear-related behavioral response (51, 62).

The amygdala has a crucial role in the modulation of attention orientation to potential threats (63, 64). Activation of β-adrenoreceptors in the BLA enhances memory consolidation associated with fear via the stimulation of the cAMP/PKA pathway (65). Lesion and agonist/antagonist studies demonstrate the critical role of the BLA in mediating the effects of stress hormones on memory consolidation of fear-related stimuli. During threat processing, the prefrontal cortex is also engaged, although more gradually than the brisk response of the amygdala, which allows flexible modulation of amygdala-based processing by providing a more detailed representation of threat attributes (63, 66). Ghosh and Chattarji (67) recently reported that targeted activation of cAMP–PKA signaling in the lateral amygdala led to generalized fear, which provides novel insight of the cellular basis in the amygdala for the alteration of emotional states from normal to pathological fear.

Naturally, the endogenous PKA inhibitor (PKI) peptide participates in the regulation of PKA by binding to the free catalytic PKA subunit, thus preventing phosphorylation of PKA targets in various tissues and cell types. In addition, PKA signaling has been investigated using pharmacological PKIs, such as the H89 and KT5720 (68). These compounds, readily crossing the cell membranes, block PKA actions through competitive inhibition of the ATP site on the PKA catalytic subunit. Also, the introduction of a non-functioning PKA mutant, such as a dominant negative version of PKA into cells has allowed researchers to perturb specific signaling through PKA and to examine PKA’s role in cell anchorage and protein expression in epithelial cells (69). Transfection of cDNA prevents binding of the R subunits to AKAPs also preventing PKA signaling and its localization to specific cellular organelles. Finally, among a number of other existing methods, PKA activity and signaling has been investigated in mice with genetic manipulation of the PKA system. These studies have allowed for particular investigation of various aspects of PKA signaling in organic systems and areas, focusing on altered physiology in intact animals. It has been anticipated that, by producing physiological changes, these mouse models might profitably be modulated for therapeutic purposes.

## Effect of Inhibition of PKA Pathway on Anxiety Behavior

Studies using inhibitors or activators of PKA helped to elucidate its role in memory formation. Inhibition of protein synthesis or PKA activity blocks LTP in the hippocampus and interferes with memory consolidation for fear in the amygdala (70–72). Also, infusion of PKIs into basolateral amygdala immediately following fear-conditioning training dose-dependently blocked consolidation of fear memory (24-h post training) but not short-term memory (4-h) (73). Infusion of inhibitor Rp-cAMP into the CEA decreased CREB function and decreased neuropeptide Y expression and provoked anxiety-like behavior and alcohol intake in non-preferring rats (74).

In addition, studies using PDE4 inhibitors help to elucidate the molecular mechanisms involved with the behavioral response (anxiolytic-like) to inhibitors of PKA, which depend in part on neurogenic action and activation of GC receptor in the hippocampus (75, 76). There is ample data to support the role of the cAMP/PKA pathway in the mediation of antidepressant/anxiolytic activity (i.e., rolipram, fluoxetine, and clozapine increase cAMP and pCREB expression) in the hippocampus; however, our understanding of the specific effects on neurogenesis is evolving.

Studies with G_s_α (*Gnas*) transgenic mice have shown that increased cAMP signaling is associated with an anxiety-like phenotype (77). Zhang et al. (78) reported that mice with reduced PDE 4B activity, the enzyme that degrades cAMP and interrupts the negative feedback of PKA pathway resulting in increased PKA activity, displayed anxiogenic behavior. In addition, transgenic mice with overexpression of the striatially enriched cAMP-producing adenyl cyclase 5 showed increased anxiety-related behavior (79). Results of the studies reviewed above indicate that increased cAMP signaling is associated with an anxiety-like phenotype, and provide indirect evidence that an increase in PKA activity may be associated with an increased risk for anxiety. Also, studies of mice with genetic deletion of specific PDE4 subtypes have reported anxiogenic behavior, suggesting that PDE4 may be involved in the regulation of anxiety (78, 80, 81).

## Effect of PKA Defects on Anxiety-Like Behavior

Prior studies in our lab showed that transgenic mice with a downregulated *Prkar1a* gene (*tTA/X2AS*, antisense transgene) (82) exhibited behavioral abnormalities, including anxiety (83) and depression. A KO mouse heterozygous for a null allele of *Prkar1a* was developed in our lab as a model to investigate Carney complex that is caused by heterozygous inactivating *PRKAR1A* mutations (84), which results in increased PKA signaling in all cells where this gene is expressed. We hypothesized that a transgenic mouse model with downregulation of *Prkar1a* would provide a research tool to evaluate the effect of altered PKA expression on anxiety-like behaviors.

In support of our hypothesis, we found that downregulation of the regulatory subunit of PKA in mice led to an augmentation of anxiety-like behavior supporting the role of PKA in modulating anxiety-like behaviors. Compared with WT mice, *Prkar1a*^+/−^ mice had higher basal and stimulated (cAMP) PKA activity levels in the central and basolateral amygdala, brain areas known to have a critical role in the processing of sensory information related to anxiety and emotion as well as regulation of arousal level (85).

Since activity in neural circuits prior to or immediately after stimuli may influence PKA activity and LTP and, therefore, may affect fear learning; we then investigated the rodent defensive response in the *Prkar1a*^+/−^ mouse hypothesizing that *Prkar1a*^+/−^ mice would exhibit an atypical response to threat detection (37, 39). As predicted, we found that in contrast to the response of WT mice, *Prkar1a*^+/−^ mice failed to exhibit behavioral changes (exploratory or defensive) to distinguish between predator versus control odor. The behavioral changes paralleled significant differences found in PKA activity between WT and *Prkar1a*^+/−^ mice in the amygdala, prefrontal cortex, and ventromedial hypothalamus (86). Our findings are consistent with results of electrophysiological studies showing that changes in amygdala circuitry and dendritic morphology affect fearful responses and correlate with BLA transmission and that the degree of anxiogenic effect of predator stress is positively associated with the degree of potentiation of amygdala circuitry (48, 87, 88). Also, since the function of the prefrontal cortex is to inhibit prepotent behavioral and promote task relevant behaviors, alterations in PKA activity in the prefrontal cortex may also have contributed to the atypical response to threat detection in the *Prkar1a*^+/−^ mice (86).

We introduced half-null alleles of *Prkaca*^+/−^ into the *Prkar1a*^+/−^ mice, hypothesizing abrogation of the excess Cα activity caused by R1α haploinsufficiency. The phenotype of *Prkaca*^+/−^ mice was characterized by attenuation but not elimination of the anxiety phenotype noted in *Prkar1a* heterozygote mice. Measurement of PKA activity in various brain areas showed increased PKA activity in the amygdala in *Prkar1a*^+/−^ compared with *Prkaca*^+/−^ or *WT*, and in part compared with *Prkar1a*^+/−^/*Prkaca*^+/−^ mice. The alteration of PKA activity in these transgenic mice was not a ubiquitous effect, since PKA activity was found to be similar between heterozygotes and WT mice in some brain areas (e.g., prefrontal cortex, hippocampus, paraventricular hypothalamus, cerebellum, and neural sensory areas). These findings highlight the importance of even modest changes in PKA activity in modulating anxiety-like behaviors and also that catalytic subunit activity is not the sole determinant of PKA’s cAMP-signaling effects (89, 90). It is also possible that compensatory mechanisms in remaining PKA subunits and PDE4 may be a factor in areas not showing any differences in PKA activity between *Prkar1a*^+/−^ and *Prkar1a*^+/−^*/Prkaca*^+/−^ and *WT* or *Prkaca*^+/−^ mice.

## Summary/Conclusion

In this review, we highlighted the association of abnormal neural processing of threat-related stimuli and anxiety disorders, which is significantly influenced by the cAMP/PKA pathway, among others. Animal models have helped to elucidate the molecular pathways that have an important role in anxiety; however, there are limitations, so cautious interpretation is appropriate. A recently developed mouse of R1a deficiency provides a unique model to investigate the direct effect of increased PKA activity on the acquisition and expression of learned fear.

Results of clinical studies support the finding that alterations in PKA and some of its substrates are associated with various psychiatric disorders, including anxiety, depression, obsessive–compulsive and bipolar disorders, schizophrenia, and panic disorder (91–97). Also, in adult patients with *PRKAR1A* mutations, we reported an increased incidence of psychiatric disorders, including anxiety, depression, and bipolar disorder (in that order), and for children with *PRKAR1A* mutations an increased incidence of learning disorders, attention deficit hyperactivity disorder, anxiety, and depression (in that order) (98). Recent animal studies support the hypothesis that selective gene intervention in the cAMP/PKA system may constitute a promising anxiolytic target.

## Author Contributions

All authors contributed equally to the writing (MK and GB) and editing (MK, GB, CS, and TW) of this manuscript.

## Disclaimer

The opinions or assertions contained herein are the private ones of the authors and are not to be construed as official or reflecting the views of the Department of Defense (DoD) or the Uniformed Services University of the Health Sciences (USUHS). NICHD, USUHS, or DOD had no further role in the study design; in the collection, analysis, and interpretation of the data; in the writing of the report, or in the decision to submit the paper for publication.

## Conflict of Interest Statement

The authors declare that the research was conducted in the absence of any commercial or financial relationships that could be construed as a potential conflict of interest.

## References

[1] Nesterova M Bossis I Wen F Horvath A Matyakhina L Stratakis CA. An immortalized human cell line bearing a PRKAR1A-inactivating mutation: effects of overexpression of the wild-type Allele and other protein kinase A subunits. J Clin Endocrinol Metab (2008) 93:565–71. 10.1210/jc.2007-1902 18056771 PMC2243228

[2] Amieux PS McKnight GS. The essential role of RI alpha in the maintenance of regulated PKA activity. Ann N Y Acad Sci (2002) 968:75–95. 10.1111/j.1749-6632.2002.tb04328.x 12119269

[3] Doskeland SO Maronde E Gjertsen BT The genetic subtypes of cAMP-dependent protein kinase – functionally different or redundant? Biochim Biophys Acta (1993) 1178:249–58. 10.1016/0167-4889(93)90201-Y 8395890

[4] Skalhegg BS Tasken K. Specificity in the cAMP/PKA signaling pathway. Differential expression, regulation, and subcellular localization of subunits of PKA. Front Biosci (2000) 5:D678–93. 10.2741/Skalhegg 10922298

[5] Gamm DM Baude EJ Uhler MD. The major catalytic subunit isoforms of cAMP-dependent protein kinase have distinct biochemical properties in vitro and in vivo. J Biol Chem (1996) 271:15736–42. 10.1074/jbc.271.26.15736 8662989

[6] Neves SR Ram PT Iyengar R. G protein pathways. Science (2002) 296:1636–9. 10.1126/science.1071550 12040175

[7] Neer EJ Heterotrimeric G proteins: organizers of transmembrane signals. Cell (1995) 80:249–57. 10.1016/0092-8674(95)90407-7 7834744

[8] Taylor SS Bubis J Toner-Webb J Saraswat LD First EA Buechler JA CAMP-dependent protein kinase: prototype for a family of enzymes. FASEB J (1988) 2:2677–85. 3294077 10.1096/fasebj.2.11.3294077

[9] Oyen O Myklebust F Scott JD Cadd GG McKnight GS Hansson V Subunits of cyclic adenosine 3’,5’-monophosphate-dependent protein kinase show differential and distinct expression patterns during germ cell differentiation: alternative polyadenylation in germ cells gives rise to unique smaller-sized mRNA species. Biol Reprod (1990) 43:46–54. 10.1095/biolreprod43.1.46 2393692

[10] Abel T Nguyen PV. Regulation of hippocampus-dependent memory by cyclic AMP-dependent protein kinase. Prog Brain Res (2008) 169:97–115. 10.1016/S0079-6123(07)00006-4 18394470 PMC2914307

[11] Liu SJ Zhang JY Li HL Fang ZY Wang Q Deng HM Tau becomes a more favorable substrate for GSK-3 when it is prephosphorylated by PKA in rat brain. J Biol Chem (2004) 279:50078–88. 10.1074/jbc.M406109200 15375165

[12] Taylor SS Buechler JA Yonemoto W. cAMP-dependent protein kinase: framework for a diverse family of regulatory enzymes. Annu Rev Biochem (1990) 59:971–1005. 10.1146/annurev.bi.59.070190.004543 2165385

[13] Bertherat J. Nuclear effects of the cAMP pathway activation in somatotrophs. Horm Res (1997) 47:245–50. 10.1159/000185471 9167959

[14] Chen C Jin N Qian W Liu W Tan X Ding F Cyclic AMP-dependent protein kinase enhances SC35-promoted Tau exon 10 inclusion. Mol Neurobiol (2014) 49:615–24. 10.1007/s12035-013-8542-3 24037441

[15] Abel T Kandel E. Positive and negative regulatory mechanisms that mediate long-term memory storage. Brain Res Brain Res Rev (1998) 26:360–78. 10.1016/S0165-0173(97)00050-7 9651552

[16] Maurice DH Ke H Ahmad F Wang Y Chung J Manganiello VC. Advances in targeting cyclic nucleotide phosphodiesterases. Nat Rev Drug Discov (2014) 13:290–314. 10.1038/nrd4228 24687066 PMC4155750

[17] Boutillier AL Gaiddon C Lorang D Roberts JL Loeffler JP. Transcriptional activation of the proopiomelanocortin gene by cyclic AMP-responsive element binding protein. Pituitary (1998) 1:33–43. 10.1023/A:1009966808106 11081181

[18] Legradi G Hannibal J Lechan RM. Pituitary adenylate cyclase-activating polypeptide-nerve terminals densely innervate corticotropin-releasing hormone-neurons in the hypothalamic paraventricular nucleus of the rat. Neurosci Lett (1998) 246:145–8. 10.1016/S0304-3940(98)00255-9 9792613

[19] Agarwal A Halvorson LM Legradi G. Pituitary adenylate cyclase-activating polypeptide (PACAP) mimics neuroendocrine and behavioral manifestations of stress: evidence for PKA-mediated expression of the corticotropin-releasing hormone (CRH) gene. Brain Res Mol Brain Res (2005) 138:45–57. 10.1016/j.molbrainres.2005.03.016 15882914 PMC1950324

[20] Stocco DM Clark BJ Regulation of the acute production of steroids in steroidogenic cells. Endocr Rev (1996) 17:221–44. 10.1210/er.17.3.221 8771357

[21] Bousquet C Chesnokova V Kariagina A Ferrand A Melmed S. cAMP neuropeptide agonists induce pituitary suppressor of cytokine signaling-3: novel negative feedback mechanism for corticotroph cytokine action. Mol Endocrinol (2001) 15:1880–90. 10.1210/mend.15.11.0733 11682619

[22] Mierke DF Giragossian C. Peptide hormone binding to G-protein-coupled receptors: structural characterization via NMR techniques. Med Res Rev (2001) 21:450–71. 10.1002/med.1018 11579442

[23] Duman RS Malberg J Nakagawa S D’Sa C. Neuronal plasticity and survival in mood disorders. Biol Psychiatry (2000) 48:732–9. 10.1016/S0006-3223(00)00935-5 11063970

[24] Dwivedi Y. Brain-derived neurotrophic factor and suicide pathogenesis. Ann Med (2010) 42:87–96. 10.3109/07853890903485730 20166812 PMC3708652

[25] Altshuler LL Casanova MF Goldberg TE Kleinman JE. The hippocampus and parahippocampus in schizophrenia, suicide, and control brains. Arch Gen Psychiatry (1990) 47:1029–34. 10.1001/archpsyc.1990.01810230045008 2241505

[26] Cotter D Mackay D Chana G Beasley C Landau S Everall IP. Reduced neuronal size and glial cell density in area 9 of the dorsolateral prefrontal cortex in subjects with major depressive disorder. Cereb Cortex (2002) 12:386–94. 10.1093/cercor/12.4.386 11884354

[27] Bremner JD Narayan M Anderson ER Staib LH Miller HL Charney DS. Hippocampal volume reduction in major depression. Am J Psychiatry (2000) 157:115–8. 10.1176/ajp.157.1.115 10618023

[28] Rajkowska G. Morphometric methods for studying the prefrontal cortex in suicide victims and psychiatric patients. Ann N Y Acad Sci (1997) 836:253–68. 10.1111/j.1749-6632.1997.tb52364.x 9616803

[29] Rosoklija G Toomayan G Ellis SP Keilp J Mann JJ Latov N Structural abnormalities of subicular dendrites in subjects with schizophrenia and mood disorders: preliminary findings. Arch Gen Psychiatry (2000) 57:349–56. 10.1001/archpsyc.57.4.349 10768696

[30] Pandey GN Dwivedi Y. Noradrenergic function in suicide. Arch Suicide Res (2007) 11:235–46. 10.1080/13811110701402587 17558608

[31] Pandey GN Dwivedi Y Ren X Rizavi HS Faludi G Sarosi A Regional distribution and relative abundance of serotonin(2c) receptors in human brain: effect of suicide. Neurochem Res (2006) 31:167–76. 10.1007/s11064-005-9006-6 16673176

[32] Dwivedi Y Pandey GN. Elucidating biological risk factors in suicide: role of protein kinase A. Prog Neuropsychopharmacol Biol Psychiatry (2011) 35:831–41. 10.1016/j.pnpbp.2010.08.025 20817068 PMC3026860

[33] Borrelli E Montmayeur JP Foulkes NS Sassone-Corsi P. Signal transduction and gene control: the cAMP pathway. Crit Rev Oncog (1992) 3:321–38. 1329990

[34] Nestler EJ Gould E Manji H Buncan M Duman RS Greshenfeld HK Preclinical models: status of basic research in depression. Biol Psychiatry (2002) 52:503–28. 10.1016/S0006-3223(02)01405-1 12361666

[35] Schacher S Castellucci VF Kandel ER. cAMP evokes long-term facilitation in aplysia sensory neurons that requires new protein synthesis. Science (1988) 240:1667–9. 10.1126/science.2454509 2454509

[36] Bourtchouladze R Abel T Berman N Gordon R Lapidus K Kandel ER. Different training procedures recruit either one or two critical periods for contextual memory consolidation, each of which requires protein synthesis and PKA. Learn Mem (1998) 5:365–74. 10454361 PMC311273

[37] Huang YY Colino A Selig DK Malenka RC. The influence of prior synaptic activity on the induction of long-term potentiation. Science (1992) 255:730–3. 10.1126/science.1346729 1346729

[38] Swanson-Park JL Coussens CM Mason-Parker SE Raymond CR Hargreaves EL Dragunow M A double dissociation within the hippocampus of dopamine D1/D5 receptor and beta-adrenergic receptor contributions to the persistence of long-term potentiation. Neuroscience (1999) 92:485–97. 10.1016/S0306-4522(99)00010-X 10408599

[39] Abel T Nguyen PV Barad M Deuel TA Kandel ER Bourtchouladze R. Genetic demonstration of a role for PKA in the late phase of LTP and in hippocampus-based long-term memory. Cell (1997) 88:615–26. 10.1016/S0092-8674(00)81904-2 9054501

[40] Vianna MR Coitinho AS Izquierdo I. Role of the hippocampus and amygdala in the extinction of fear-motivated learning. Curr Neurovasc Res (2004) 1:55–60. 10.2174/1567202043480170 16181066

[41] Robinson-White A Stratakis CA. Protein kinase A signaling: “cross-talk” with other pathways in endocrine cells. Ann N Y Acad Sci (2002) 968:256–70. 10.1111/j.1749-6632.2002.tb04340.x 12119281

[42] Konradi C Cole RL Heckers S Hyman SE. Amphetamine regulates gene expression in rat striatum via transcription factor CREB. J Neurosci (1994) 14:5623–34. 8083758 10.1523/JNEUROSCI.14-09-05623.1994 PMC6577107

[43] Charmandari E Kino T Souvatzoglou E Chrousos GP. Pediatric stress: hormonal mediators and human development. Horm Res (2003) 59:161–79. 10.1159/000069325 12649570

[44] Kino T De Martino MU Charmandari E Mirani M Chrousos GP. Tissue glucocorticoid resistance/hypersensitivity syndromes. J Steroid Biochem Mol Biol (2003) 85:457–67. 10.1016/S0960-0760(03)00218-8 12943736

[45] McEwen BS Protective and damaging effects of stress mediators: the good and bad sides of the response to stress. Metabolism (2002) 51:2–4. 10.1053/meta.2002.33183 12040533

[46] Charney DS. Psychobiological mechanisms of resilience and vulnerability: implications for successful adaptation to extreme stress. Am J Psychiatry (2004) 161:195–216. 10.1176/appi.ajp.161.2.195 14754765

[47] Kalin NH Shelton SE Davidson RJ. The role of the central nucleus of the amygdala in mediating fear and anxiety in the primate. J Neurosci (2004) 24:5506–15. 10.1523/JNEUROSCI.0292-04.2004 15201323 PMC6729317

[48] Adamec R Shallow T Burton P Anxiolytic and anxiogenic effects of kindling – role of baseline anxiety and anatomical location of the kindling electrode in response to kindling of the right and left basolateral amygdala. Behav Brain Res (2005) 159:73–88. 10.1016/j.bbr.2004.10.004 15795000

[49] Blanchard DC Griebel G Pobbe R Blanchard RJ. Risk assessment as an evolved threat detection and analysis process. Neurosci Biobehav Rev (2011) 35:991–8. 10.1016/j.neubiorev.2010.10.016 21056591

[50] Wood SJ Toth M. Molecular pathways of anxiety revealed by knockout mice. Mol Neurobiol (2001) 23:101–19. 10.1385/MN:23:2-3:101 11817214

[51] Maren S Quirk GJ. Neuronal signalling of fear memory. Nat Rev Neurosci (2004) 5:844–52. 10.1038/nrn1535 15496862

[52] Phelps EA LeDoux JE. Contributions of the amygdala to emotion processing: from animal models to human behavior. Neuron (2005) 48:175–87. 10.1016/j.neuron.2005.09.025 16242399

[53] Davis M Shi C The amygdala. Curr Biol (2000) 10:R131 10.1016/S0960-9822(00)00345-6 10704422

[54] LeDoux JE Iwata J Cicchetti P Reis DJ. Different projections of the central amygdaloid nucleus mediate autonomic and behavioral correlates of conditioned fear. J Neurosci (1988) 8:2517–29. 2854842 10.1523/JNEUROSCI.08-07-02517.1988 PMC6569498

[55] Knight DC Nguyen HT Bandettini PA. The role of the human amygdala in the production of conditioned fear responses. Neuroimage (2005) 26:1193–200. 10.1016/j.neuroimage.2005.03.020 15961053

[56] Holland PC Gallagher M. Amygdala circuitry in attentional and representational processes. Trends Cogn Sci (1999) 3:65–73. 10.1016/S1364-6613(98)01271-6 10234229

[57] Rollins BL Stines SG McGuire HB King BM. Effects of amygdala lesions on body weight, conditioned taste aversion, and neophobia. Physiol Behav (2001) 72:735–42. 10.1016/S0031-9384(01)00433-4 11337006

[58] Wright CI Martis B Schwartz CE Shin LM Fischer HH McMullin K Novelty responses and differential effects of order in the amygdala, substantia innominata, and inferior temporal cortex. Neuroimage (2003) 18:660–9. 10.1016/S1053-8119(02)00037-X 12667843

[59] Rauch SL Shin LM Phelps EA Neurocircuitry models of posttraumatic stress disorder and extinction: human neuroimaging research – past, present, and future. Biol Psychiatry (2006) 60:376–82. 10.1016/j.biopsych.2006.06.004 16919525

[60] Shin LM Rauch SL Pitman RK. Amygdala, medial prefrontal cortex, and hippocampal function in PTSD. Ann N Y Acad Sci (2006) 1071:67–79. 10.1196/annals.1364.007 16891563

[61] Yang RJ Mozhui K Karlsson RM Cameron HA Williams RW Holmes A. Variation in mouse basolateral amygdala volume is associated with differences in stress reactivity and fear learning. Neuropsychopharmacology (2008) 33:2595–604. 10.1038/sj.npp.1301665 18185497

[62] Fanselow MS LeDoux JE Why we think plasticity underlying Pavlovian fear conditioning occurs in the basolateral amygdala. Neuron (1999) 23:229–32. 10.1016/S0896-6273(00)80775-8 10399930

[63] LeDoux JE Emotion circuits in the brain. Annu Rev Neurosci (2000) 23:155–84. 10.1146/annurev.neuro.23.1.155 10845062

[64] Davis M Whalen PJ. The amygdala: vigilance and emotion. Mol Psychiatry (2001) 6:13–34. 10.1038/sj.mp.4000812 11244481

[65] Ferry B Roozendaal B McGaugh JL. Basolateral amygdala noradrenergic influences on memory storage are mediated by an interaction between beta- and alpha1-adrenoceptors. J Neurosci (1999) 19:5119–23. 10366644 10.1523/JNEUROSCI.19-12-05119.1999 PMC6782651

[66] Brotman MA Rich BA Schmajuk M Reising M Monk CS Dickstein DP Attention bias to threat faces in children with bipolar disorder and comorbid lifetime anxiety disorders. Biol Psychiatry (2007) 61:819–21. 10.1016/j.biopsych.2006.08.021 17338904

[67] Ghosh S Chattarji S. Neuronal encoding of the switch from specific to generalized fear. Nat Neurosci (2015) 18:112–20. 10.1038/nn.3888 25436666

[68] Murray AJ. Pharmacological PKA inhibition: all may not be what it seems. Sci Signal (2008) 1:re4. 10.1126/scisignal.122re4 18523239

[69] Hayashida K Johnston DR Goldberger O Park PW. Syndecan-1 expression in epithelial cells is induced by transforming growth factor beta through a PKA-dependent pathway. J Biol Chem (2006) 281:24365–74. 10.1074/jbc.M509320200 16807246

[70] Parsons RG Davis M. A metaplasticity-like mechanism supports the selection of fear memories: role of protein kinase a in the amygdala. J Neurosci (2012) 32:7843–51. 10.1523/JNEUROSCI.0939-12.2012 22674260 PMC3375025

[71] Zhao WQ Polya GM Wang BH Gibbs ME Sedman GL Ng KT. Inhibitors of cAMP-dependent protein kinase impair long-term memory formation in day-old chicks. Neurobiol Learn Mem (1995) 64:106–18. 10.1006/nlme.1995.1049 7582818

[72] Matthies H Reymann KG. Protein kinase A inhibitors prevent the maintenance of hippocampal long-term potentiation. Neuroreport (1993) 4:712–4. 10.1097/00001756-199306000-00028 8347813

[73] Schafe GE Atkins CM Swank MW Bauer EP Sweatt JD LeDoux JE. Activation of ERK/MAP kinase in the amygdala is required for memory consolidation of Pavlovian fear conditioning. J Neurosci (2000) 20:8177–87. 11050141 10.1523/JNEUROSCI.20-21-08177.2000 PMC6772720

[74] Pandey SC Zhang H Roy A Xu T. Deficits in amygdaloid cAMP-responsive element-binding protein signaling play a role in genetic predisposition to anxiety and alcoholism. J Clin Invest (2005) 115:2762–73. 10.1172/JCI24381 16200210 PMC1236671

[75] Li YF Huang Y Amsdell SL Xiao L O’Donnell JM Zhang HT. Antidepressant- and anxiolytic-like effects of the phosphodiesterase-4 inhibitor rolipram on behavior depend on cyclic AMP response element binding protein-mediated neurogenesis in the hippocampus. Neuropsychopharmacology (2009) 34:2404–19. 10.1038/npp.2009.66 19516250 PMC2743762

[76] Anacker C Zunszain PA Cattaneo A Carvalho LA Garabedian MJ Thuret S Antidepressants increase human hippocampal neurogenesis by activating the glucocorticoid receptor. Mol Psychiatry (2011) 16:738–50. 10.1038/mp.2011.26 21483429 PMC3121947

[77] Favilla C Abel T Kelly MP. Chronic Galphas signaling in the striatum increases anxiety-related behaviors independent of developmental effects. J Neurosci (2008) 28:13952–6. 10.1523/JNEUROSCI.4986-08.2008 19091983 PMC2688724

[78] Zhang HT Huang Y Masood A Stolinski LR Li Y Zhang L Anxiogenic-like behavioral phenotype of mice deficient in phosphodiesterase 4B (PDE4B). Neuropsychopharmacology (2008) 33:1611–23. 10.1038/sj.npp.1301537 17700644 PMC2728355

[79] Kim KS Lee KW Baek IS Lim CM Krishnan V Lee JK Adenylyl cyclase-5 activity in the nucleus accumbens regulates anxiety-related behavior. J Neurochem (2008) 107:105–15. 10.1111/j.1471-4159.2008.05592.x 18673448 PMC2744302

[80] McGirr A Lipina TV Mun HS Georgiou J Al-Amri AH Ng E Specific inhibition of phosphodiesterase-4B results in anxiolysis and facilitates memory acquisition. Neuropsychopharmacology (2016) 41:1080–92. 10.1038/npp.2015.240 26272049 PMC4748432

[81] Hansen RT III Conti M Zhang HT. Mice deficient in phosphodiesterase-4A display anxiogenic-like behavior. Psychopharmacology (Berl) (2014) 231:2941–54. 10.1007/s00213-014-3480-y 24563185

[82] Griffin KJ Kirschner LS Matyakhina L Stergiopoulos S Robinson-White A Lenherr S Down-regulation of regulatory subunit type 1A of protein kinase A leads to endocrine and other tumors. Cancer Res (2004) 64:8811–5. 10.1158/0008-5472.CAN-04-3620 15604237

[83] Batista DL Weinberg F Stergiopoulos SG Meoli E Griffin K Stratakis CA, editors. Behavior Modifications in Mice Expressing R1 Alpha Protein Kinase A Subunit Mutant. San Diego, CA: Endocrine Society (2005).

[84] Kirschner LS Kusewitt DF Matyakhina L Towns WH II Carney JA Westphal H A mouse model for the Carney complex tumor syndrome develops neoplasia in cyclic AMP-responsive tissues. Cancer Res (2005) 65:4506–14. 10.1158/0008-5472.CAN-05-0580 15930266

[85] Keil MF Briassoulis G Gokarn N Nesterova M Wu TJ Stratakis CA. Anxiety phenotype in mice that overexpress protein kinase A. Psychoneuroendocrinology (2012) 37:836–43. 10.1016/j.psyneuen.2011.09.016 22024111 PMC3320692

[86] Keil MF Briassoulis G Nesterova M Miraftab N Gokarn N Wu TJ Threat bias in mice with inactivating mutations of Prkar1a. Neuroscience (2013) 241:206–14. 10.1016/j.neuroscience.2013.03.027 23531435 PMC3646976

[87] Vyas A Pillai AG Chattarji S. Recovery after chronic stress fails to reverse amygdaloid neuronal hypertrophy and enhanced anxiety-like behavior. Neuroscience (2004) 128:667–73. 10.1016/j.neuroscience.2004.07.013 15464275

[88] Adamec R Blundell J Burton P. Anxiolytic effects of kindling role of anatomical location of the kindling electrode in response to kindling of the right basolateral amygdala. Brain Res (2004) 1024:44–58. 10.1016/j.brainres.2004.06.074 15451366

[89] Briassoulis G Keil MF Naved B Nesterova M Gokarn N Wu TJ, editors. Role of the Catalytic Subunit in Behavioral Phenotype of Mice with Inactivating Mutations of PRKAR1A or PRKACA. Society for Neuroscience. Washington, DC: Hormone and Metabolic Research (2012).

[90] Briassoulis G Keil MF Naved B Liu S Starost MF Nesterova M Studies of mice with cyclic AMP-dependent protein kinase (PKA) defects reveal the critical role of PKA’s catalytic subunits in anxiety. Behav Brain Res (2016). 10.1016/j.bbr.2016.03.001 PMC4853257 26992826

[91] Chang A Li PP Warsh JJ. cAMP-dependent protein kinase (PKA) subunit mRNA levels in postmortem brain from patients with bipolar affective disorder (BD). Brain Res Mol Brain Res (2003) 116:27–37. 10.1016/S0169-328X(03)00211-0 12941458

[92] Dell’Osso L Carmassi C Palego L Trincavelli ML Tuscano D Montali M Serotonin-mediated cyclic AMP inhibitory pathway in platelets of patients affected by panic disorder. Neuropsychobiology (2004) 50:28–36. 10.1159/000077938 15179017

[93] Perez J Tardito D Racagni G Smeraldi E Zanardi R. Protein kinase A and Rap1 levels in platelets of untreated patients with major depression. Mol Psychiatry (2001) 6:44–9. 10.1038/sj.mp.4000795 11244484

[94] Perez J Tardito D Mori S Racagni G Smeraldi E Zanardi R. Abnormalities of cyclic adenosine monophosphate signaling in platelets from untreated patients with bipolar disorder. Arch Gen Psychiatry (1999) 56:248–53. 10.1001/archpsyc.56.3.248 10078502

[95] Perez J Tardito D Ravizza L Racagni G Mori S Maina G. Altered cAMP-dependent protein kinase A in platelets of patients with obsessive-compulsive disorder. Am J Psychiatry (2000) 157:284–6. 10.1176/appi.ajp.157.2.284 10671404

[96] Tardito D Maina G Tura GB Bogetto F Pioli R Ravizza L The cAMP-dependent protein kinase substrate Rap1 in platelets from patients with obsessive compulsive disorder or schizophrenia. Eur Neuropsychopharmacol (2001) 11:221–5. 10.1016/S0924-977X(01)00088-8 11418282

[97] Tardito D Tura GB Bocchio L Bignotti S Pioli R Racagni G Abnormal levels of cAMP-dependent protein kinase regulatory subunits in platelets from schizophrenic patients. Neuropsychopharmacology (2000) 23:216–9. 10.1016/S0893-133X(99)00161-X 10882848

[98] Keil MF Lyssikatos C Shaikh M Belyavskaya E Elliott B Batista D, editors. Effects of PRKAR1A Mutations in Behavior and Brain Function. European Congress of Endocrinology. Wroclaw: Endocrine Abstracts (2014).

